# Prevention of Congenital Syphilis Through Antenatal Screenings in Lusaka, Zambia: A Systematic Review

**DOI:** 10.7759/cureus.2078

**Published:** 2018-01-16

**Authors:** Faisal Akhtar, Sabah Rehman

**Affiliations:** 1 Neurology, Ochsner Health System; 2 Biochemistry, Shifa College Of Medicine

**Keywords:** prevention, neonatal, disease prevention, congenital syphilis, antenatal care, healthcare burden, healthcare cost reduction, healthcare policy, prenatal care

## Abstract

Congenital syphilis is one of the preventable diseases caused by the gram-negative bacteria Treponema pallidum; yet, it imposes a serious global health and economic burden, with more than half of the cases resulting in serious adverse outcomes, including infant mortality. Mother-to-child transmission (MTCT) of syphilis is estimated at 3.6 million adjusted life years (DALYs) and around $309 million in medical costs. In 2006, an estimated 9.7 million children of age less than five years died in developing countries; almost four million were neonatal deaths. There were 3.2 million stillbirths globally, among whom 95% were in the developing countries. In sub-Saharan Africa, there is an estimated 2.7% (0.1%-10.3%) of pregnant women infected with syphilis, representing more than 900,000 pregnancies at risk each year. There were many non-specific and specific diagnostic tests used in the past, which required laboratory equipment and electricity, but there are many newer tests available now that provide rapid results with high sensitivity and specificity, e.g., the immunochromatographic strip (ICS) and rapid syphilis tests (RST). Early syphilis can be completely eliminated with a single injection of penicillin, which is readily available, cheap, and highly effective, and treating pregnant women with penicillin is 98% effective at preventing congenital syphilis. Targeting women at a high risk of having syphilis makes universal screening in antenatal programs the most efficacious way to prevent syphilis-associated morbidity and mortality. The potential for a program to prevent congenital syphilis in the perinatal, neonatal, and postnatal periods is evident. While considering resource allocation to child survival programs in areas where the prevalence of syphilis is high, officials need to include antenatal syphilis screening, using rapid tests and treatment at the first contact of the mother with the health care system. In countries like Zambia and other resource-limited settings, a same-day test and treatment with penicillin should be prioritized to achieve the goal of eliminating congenital syphilis. Eliminating MTCT of syphilis through screening and treatment in antenatal care (ANC) is highly cost-effective in a wide range of settings, especially in countries with a high prevalence.

## Introduction and background

Congenital syphilis is a preventable disease; yet, its prevalence in resource-poor countries is significantly high, along with the adverse outcomes associated with the disease. Antenatal screening is one of the most effective ways to address this issue, but it is also one of the biggest public health challenges. So, to address the issue of whether antenatal screening is one of the best options available for prevention of the disease, let's first look into the background of the disease, the global health burden, and the burden in resource-poor countries, especially African countries, including Zambia.

a. Background of syphilis

Syphilis is caused by the gram-negative bacteria Treponema pallidum. It is further divided into the primary, secondary, and tertiary stages based on the signs and symptoms of the disease. Infection of the fetus from the infected mother results in congenital syphilis. In the first four years of acquiring syphilis, untreated women have a 70% chance of transmitting the infection to their fetus. If untreated for syphilis, 40% of these pregnancies results in perinatal death. Even the live born neonates are infected and can develop acute systemic illness, bone deformities, developmental disabilities, blindness, or deafness. Fifty percent of the infected neonates will manifest these problems immediately, whereas others will develop these later in their life. There is strong evidence indicating that syphilis, like other causes of genital ulcers, enhances human immunodeficiency virus (HIV) transmission, which makes it more important to prevent syphilis for controlling HIV.

b. Available tests for congenital syphilis

Syphilis can be diagnosed based on non-specific tests like rapid plasma reagin (RPR) and venereal disease research laboratory (VDRL). Specific tests include indirect fluorescent treponemal antibody absorption test (FTA-ABS). There are many advantages and disadvantages to both tests. Recently, there have been many more tests available for identifying the disease effectively and efficiently.

Syphilis is a persistent public health issue in many low-income countries that have limited capacity for testing and rely mostly on the non-Treponema test (high sensitivity) and a specific Treponema testing (high specificity). However, the recent development of a new rapid Treponema test can scale up the screening process in setups where traditional tests are unavailable for any reason. In studies conducted by Tucker et al., ICS syphilis tests have a high sensitivity (median 0·86, interquartile range 0·75–0·94) and a higher specificity (0·99, 0·98–0·99), both comparable with non-Treponema screening test characteristics, suggesting further research evaluating ICS syphilis tests among primary syphilis cases and among patients infected with HIV for the effective roll-out of syphilis screening programs. ICS syphilis tests are simple, inexpensive, and do not need to be refrigerated or need highly trained laboratory personnel for carrying out the test [1].

Another study by Kuznik et al. highlighted the importance of point-of-care syphilis testing, which has high accuracy, ease of use, and can potentially increase the coverage of antenatal screening. They also found the use of ICS tests for antenatal screening for syphilis to be highly cost-effective in Sub-Saharan African countries, with a substantial reduction in disability-adjusted life years (DALYs) [2].

An assessment by Bonawitz et al. found that the recently developed rapid syphilis tests (RST) have a high sensitivity (85.7%-100%) and specificity (96%-100%), and these tests do not require the traditional laboratory infrastructure required for carrying out the rapid plasma reagin (RPR) test [3].

A cohort study by Owusu-Edusei et al., of 1000 pregnant women in a highly syphilis prevalent population, developed a model based on a comparison of the health and economic outcomes of traditional testing for syphilis - rapid plasma reagin anti-Treponema pallidum assay (RPR-TPHA) - and new tests - dual point of care (dual-POC) and ICS and found out that the ICS strategy was the most cost-saving one followed by the dual-POC strategy [4].

Kuznik et al. reported that immunochromatographic point-of-care tests are rapid, reliable, inexpensive, and highly cost-effective within antenatal care (ANC) settings [5].

Terris-Prestholt et al. highlighted the importance of time constraints related to the traditional RPR test, where tests were usually done in batches and given the next day or so. They found that the majority of the women never came for picking up the test results, even if they were positive, lacking the usefulness of RPR in ANC and further highlighting the importance of rapid syphilis immunochromatographic strips (ICS) and dual rapid syphilis tests (RST) [6].

c. Treatment options for congenital syphilis

Since there is no nonhuman host for syphilis, its elimination is biologically feasible. Further, the serological tests for diagnosis are also relatively accurate (> 95% sensitivity and specificity). Early syphilis can be completely eliminated with a single injection of penicillin. The treatment of syphilis in seropositive pregnant women is one dose of intramuscular (IM) penicillin at least 30 days prior to delivery, which also reduces the risk of adverse pregnancy outcome, like a non-infected mother, even though the full treatment of latent maternal syphilis requires three doses of IM penicillin [3].

A meta-analysis by Kuznik et al. concluded that the adverse outcomes of pregnancy associated with syphilis can be prevented if the infected mothers are identified and treated before the third trimester [5].

Bowen et al. found that in pregnant women with syphilis who deliver after 20 weeks of gestation, treatment with penicillin is 98% effective at preventing congenital syphilis [7]. Penicillin is readily available, cheap, and highly effective against Treponema pallidum [8].

d. Global burden

Syphilis during pregnancy imposes a serious global health and economic burden, with more than half of the cases resulting in serious adverse outcomes, including infant mortality. Mother-to-child transmission (MTCT) of syphilis is estimated at 3.6 million disability-adjusted life years (DALYs) and around $309 million in medical costs [9].

In 2004, World Health Organization (WHO) estimated that the number of cases of congenital syphilis ranged from 700,000 to 1.5 million and estimated a 45% to 70% probability of vertical transmission. This accounted for an estimated 420,000 to 600,000 perinatal deaths, up to 40% resulting in stillbirth and 20% in neonatal deaths [10].

World Bank ranks syphilis fifth globally in terms of disability-adjusted life days (DALDs) lost per capita per year. Centers for disease control and prevention (CDC) estimated a loss of 16 DALDs per capita per year in the developing world. Estimated syphilis-associated disability-adjusted life years (DALYs) lost among children aged < 5 years are 500,000 per year [2].

WHO estimates two million pregnant women with active syphilis each year, most of whom are from low-income and middle-income countries. Sixty-nine percent of untreated or inadequately treated pregnant women result in adverse pregnancy outcomes. These untreated pregnancies can result in either late abortion or stillbirth in 25% of the cases, with prematurity or low birthweight in 13%, neonatal death in 11%, and infected syphilitic infant in 20% [11].

The trends of congenital syphilis in the United States showed that the rate of congenital syphilis decreased during 1991-2005 but increased slightly during 2005-2008. There was an overall decreased rate from 10.5 to 8.4 cases per 100,000 live births during 2008-2012, and then an increased rate of 11.6 cases per 100,000 live births in 2014. There was an increased rate of congenital syphilis across all regions of the US, primarily due to a 22% national increase in the rate of primary and secondary types of syphilis among women during the same period [7].

e. Burden in Africa or resource-poor countries

In the 1980s, Africa was facing the problems associated with sexually transmitted diseases like syphilis and gonorrhea during pregnancy in similar severity and magnitude to those faced by the Western world during the early 1900s. There was a reported prevalence of syphilis among pregnant women in Africa attending the ANC ranging from 4% to 15% [12].

There were an estimated 500,000 perinatal deaths per year in sub-Saharan Africa from syphilis. In 2006, an estimated 9.7 million children aged < 5 years died in developing countries; almost four million were neonatal deaths. There were 3.2 million stillbirths globally, among whom 95% were in developing countries [10].

In sub-Saharan Africa, an estimated 2.7% of pregnant women (0.1%-10.3%) are infected with syphilis, representing more than 900,000 pregnancies at risk each year. It is an important public health problem, causing significant perinatal morbidity and mortality in these countries. In a recent meta-analysis, comparing the risk of adverse pregnancy outcomes between untreated maternal syphilis and non-syphilis pregnancy, an adverse outcome risk of 53.4% to 81.8% was seen in untreated maternal syphilis versus 10.2% to 20.8% in women without syphilis [5].

f. Preparation of adverse outcomes in these countries

The attributable adverse pregnancy outcomes from syphilis pose a serious public health concern in developing countries, with a reported prevalence of syphilis among pregnant women in sub-Saharan Africa ranging from 3% to 17% [4].

In all sub-Saharan African countries, an estimated incidence of adverse pregnancy outcomes accounted for 205,901 per year, including stillbirths, which were 88,376, neonatal deaths in 34,959 cases, and low birth weight in 22,483 cases. Congenital syphilis was reported in 60,084 cases, which resulted in 12.5 million DALYs [5].

Looking at the historical background of congenital syphilis, ease of diagnosis and treatment options are available; yet, a substantial disease burden of the disease exists especially in Africa, including Zambia and other resource-limited countries. This raises a question on why there have been no successful strategies formulated and implemented by the world of public health to curtail the disease. So, the research question for this study logically is "Antenatal screening for congenital syphilis: An answer or a healthcare burden in low-income countries like Zambia."

## Review

Methods

For this systematic review, the resource search engines of Tulane University Matas Library of the Health Sciences were used. Search for studies on the epidemiology, clinical aspects, prevention, and management of congenital syphilis was mainly from PubMed and Embase, and a few Google Scholars’ articles were considered. Publications by WHO and the CDC and reference lists from review articles and book chapters were also searched. We also relied on our own knowledge of current issues in the ﬁeld, referencing articles that, in our opinion, represent important contributions.

Keywords used were ‘congenital syphilis,’ ‘antenatal,’ ‘prenatal,’ ‘screening,’ ‘Africa,’ ‘Zambia,’ ‘epidemiology,’ ‘prevention,’ and ‘therapy.’ No limits were set for search criteria, although papers published after 1995 have been preferentially included. All those articles that were not published in English were excluded. The initial count of articles was close to 100; the abstracts of those articles were read and shortlisted to 25, which were read thoroughly. In the result section, only those articles were finally selected that had fulfilled the following inclusion criteria.

a. Relevance of the study or article to health professionals caring for pregnant women, infants, and children in African countries b. Detailed statistical analysis provided

Results

The results of this systematic review are displayed in Table 1, which lists the final six articles and their statistical relevance.

**Table 1:** Articles and their statistical relevance SSA: Sub-Saharan Africa; DALY: disability-adjusted life year; ICS: immunochromatographic strip; RST: rapid syphilis test; dual-POC: dual nontreponemal/treponemal point-of-care test; ANC: antenatal care

Title/Author	Main research Goals	Study Design	Primary Statistical findings	Conclusions
Antenatal Syphilis Screening Using Point-of-Care Testing in Sub-Saharan African Countries: A Cost-Effectiveness Analysis [2].	To evaluate the cost-effectiveness and budget impact of antenatal syphilis screening for 43 countries in SSA and estimate the impact of universal screening on stillbirths, neonatal deaths, congenital syphilis, and disability-adjusted life years (DALYs) averted.	Decision analytic model: Average cost/DALY averted in US $ (95% CI) Prevalence target rate (range)	$11 ($5-$77) 0.038% (0.002%-0.113%)	Universal antenatal screening of pregnant women in clinics may reduce the annual number of stillbirths by up to 64,000, neonatal deaths by up to 25,000, and annual incidence of congenital syphilis by up to 32,000, and avert up to 2.6 million DALYs at an estimated annual direct medical cost of US$20.8 million. Use of ICS tests for antenatal syphilis screening is highly cost-effective in SSA. Substantial reduction in DALYs can be achieved at a relatively modest budget impact. In SSA, antenatal programs should expand access to syphilis screening using the ICS test.
Assessment of the impact of rapid syphilis tests on syphilis screening and treatment of pregnant women in Zambia [3].	To evaluate the impact of rapid syphilis tests (RSTs) on syphilis testing and treatment in pregnant women in Kalomo District, Zambia.	Quasi-experimental evaluation design with baseline, midline, and end line comparisons.	The proportion of women screened improved from baseline (140/1365, 10.6%) to midline (976/1446, 67.5%), finally decreasing at end line (752/1337, 56.3%) (P b 0.001). No significant difference in the proportion of syphilis-seroreactive pregnant women who received 1 dose of penicillin before (1/2, 50%) or after (5/48, 10.4%; P = 0.199)	With RST scale-up in Zambia and other resource-limited settings, same-day test and treatment with penicillin should be prioritized to achieve the goal of eliminating congenital syphilis.
Cost-Effectiveness of a Dual Non-Treponemal/Treponemal Syphilis Point-of-Care Test to Prevent Adverse Pregnancy Outcomes in Sub-Saharan Africa [4].	To compare the health and economic outcomes of dual nontreponemal/treponemal point-of-care test (Dual-POC) that simultaneously detects both nontreponemal and treponemal antibodies with existing syphilis tests/testing algorithms in a high prevalence setting.	N/A	N/A	The dual-POC test may help save cost in resource poor settings where disease prevalence (and loss to follow-up) is high, while substantially reducing overtreatment.
Effectiveness of interventions to improve screening for syphilis in pregnancy: a systematic review and meta-analysis [11].	To review the literature systematically to determine the effectiveness of screening interventions to prevent congenital syphilis and other adverse pregnancy outcomes.	A systematic review and meta-analysis Pooled RR (95% CI) Perinatal death Stillbirth	0.46 (0.26-0.82) 0.42 (0.19-0.93)	Interventions to improve the coverage and effect of screening programs for antenatal syphilis could reduce the syphilis-attributable incidence of stillbirth and perinatal death by 50%.
Estimating the Public Health Burden Associated with Adverse Pregnancy Outcomes Resulting from Syphilis Infection Across 43 Countries in Sub-Saharan Africa [5].	To estimate the public health burden resulting from adverse pregnancy outcomes due to syphilis infection among pregnant women not screened for syphilis in 43 countries in sub-Saharan Africa.	Looked at Annual no of live births based on a 2012 data reported by UN's Children Fund. Calculated incidence (95% CI) of adverse outcomes Stillbirth Neonatal death Low birth weight Congenital syphilis And DALYs	88,376 (60,854-121,713) 34,959 (23,330-50,076) 22,483 (0-98,847) 60,084 (29,073-112,414) 12.5 Million DALYs	Substantial infant mortality and morbidity results from maternal syphilis infection concentrated in countries with low access to ANC or low rates of syphilis screening.
Syphilis-associated perinatal and infant mortality in rural Malawi [6].	Compared with non-syphilitic women, those with active syphilis were more likely to experience stillbirths as well as the early and late neonatal deaths and even postnatal deaths of their children.	Prospective study design Univariate analysis-OR (95% CI) Fetal loss Stillbirth Neonatal death Perinatal death Postnatal death Infant death	8.81 (5.41-14.29) 11.01 (6.54-18.49) 4.5 (2.43-8.23) 8.36 (5.38-12.94) 2.41 (1.30-4.40) 3.29 (2.05-5.26)	The potential for a program to prevent congenital syphilis in the perinatal, neonatal, and postnatal periods is evident. In considering resource allocation to child survival programs in areas where the prevalence of syphilis is high, officials need to include antenatal syphilis screening, using rapid tests and treatment at the first contact of the mother with the health care system.

Discussion

Based on the studies analyzed, one thing found common was that syphilis (congenital syphilis) is preventable and treatable, and antenatal screening is a great tool to achieve this target. (In a few studies, they found that there was a mismatch between the actual syphilis burden and the diagnostic capacity in many regions, which halted the efforts to meet syphilis control guidelines, which further calls for the widespread screening of syphilis [1].)

One of the key point or issues to start any intervention is to analyze the cost-effectiveness of that program. All the studies conducted in the sub-Saharan African countries demonstrated that syphilis screening is highly cost-effective. A study by Kuznik et al. (2013) in all 43 sub-Saharan African countries demonstrated an average cost/DALY averted of US$ 11, which also showed that screening remains highly cost-effective even if the current prevalence rate falls from 3.1% to 0.038% [2].

There was a low cost associated with the screening; however, in a few studies, it was shown that the diagnosis of syphilis was made solely based on history and clinical presentation, especially in resource-poor settings, to mitigate the cost of screening during the antenatal visits. This can cause a false negative or even false positive diagnosis. This approach is certainly not going to be beneficial either in the short or the long term; instead, universal on-site syphilis screening of all pregnant women at their first antenatal visit is the most appropriate approach [10].

This approach is also beneficial in preventing fetal deaths and morbidity due to congenital syphilis since the identification and treatment of the disease by the middle of the second trimester proved the most beneficial in terms of reducing adverse pregnancy outcomes. Mandatory screening for syphilis also answers the question that syphilis remains asymptomatic in many women, so clinical diagnosis cannot be totally correct [13].

The WHO global initiative to eliminate congenital syphilis also recommends many strategies that highlight reducing the overall prevalence of syphilis in adults and providing family planning services, access to high-quality antenatal care for all pregnant women, and syphilis screening and treatment options within the antenatal care services. Adopting these strategies can have a profound effect on reducing the adverse pregnancy outcomes caused by syphilis. Hawkes et al. (2011) also concluded that the coverage and effect of screening programs for antenatal syphilis could reduce the syphilis-attributable incidence of stillbirth and perinatal death by 50% [11].

The study by Kuznik et al. (2015) of all 43 sub-Saharan African countries showed that in the majority of the countries with low access to ANC or low rates of syphilis screening, there is substantial infant mortality and morbidity seen in those countries, which further emphasizes the importance of antenatal screening programs to avoid such adverse outcomes [5].

Schulz et al. found that with the advent of newer, better, and more rapid tests, researchers in Zambia analyzed the rapid plasma reagin (RPR) test and found that it has more false negative rates than false positive, which can actually account for an underestimation of the actual prevalence of syphilis in Zambia. This highlights the importance of administrating newer and better tests in ANCs [12].

Jenniskens et al. (1995) showed that a decentralized syphilis control program in pregnant women has also demonstrated some promising results when implemented in many African countries. Pregnant women were screened and treated at the same visit if they were found seroreactive, and were also counseled on the importance of partner treatment to protect their unborn babies from congenital syphilis. The results were appreciable since the problem with the conventional testing was that they were mostly sent in batches and the majority of women never showed up again to collect the result, which nullifies the importance of the screening in the first place. Even the total cost incurred per prevented case was US$ 50, which demonstrated that such projects are feasible and inexpensive to operate in such settings [14].

One of the issues which needs to be addressed is educating people on the importance of prenatal screening for syphilis in reducing the adverse outcomes during pregnancy. The study by Hira et al. in Lusaka, Zambia, on 150 pregnant women for one year, demonstrated the importance of health education to improve the early attendance to the antenatal clinics and a reduction in the adverse outcomes of pregnancy. The on-site screening and treatment of the seroreactive women, and their partners, for syphilis   were carried out, which resulted in a reduction of the adverse outcomes of pregnancy to 28.3%, as compared to 72.4%. The average cost of each prenatal screening was US$ 0.60 and for averting each adverse outcome, it was US$ 12 [15].

Another study by McDermott et al. (1993) demonstrated the importance of universal screening in antenatal programs as the most efficacious way to prevent syphilis-associated morbidity and mortality, which can prevent syphilis-associated stillbirths and perinatal, neonatal, and postnatal deaths [16].

Limitations

a. Most of the literature available on Lusaka, Zambia is older literature, directing toward the lack of interest in preventing syphilis in this country. b. Asymptomatic presentation of the disease. c. Lack of international interest-shown by the cost of screening and intervention and no reduction in the overall rates of the disease, demonstrating the lack of interest of the international community. d. Treating infected pregnant women with penicillin can prevent congenital syphilis; however, the genital sores of primary syphilis are painless and mostly go unnoticed by the majority of women, who do not seek care. e. There are many fetal deaths a year from congenital syphilis, even though prevention by antenatal screening is cost-effective, rather cost-saving, pinpointing the lack of international and national appreciation of the burden of congenital syphilis and insufficient political will to provide effective antenatal screening programs in the areas needed. f. Even most African countries do have policies for antenatal syphilis screening in place for decades, but they lack implementation, resulting in fewer than one in eight of all pregnant women estimated to get screened for syphilis, despite the low costs of both screening and treatment. g. The rapid reagin serological test is surely one of the cheapest options, but the need for electricity reduces its effectiveness since electricity is not available all the time in many rural health facilities in Africa. h. Many towns in African countries are operationally ineffective because of the centralized testing and testing in batches, which results in low follow-up and treatment of women who have tested positive.

## Conclusions

Looking at all the results from the selected studies and the literature search conducted, the conclusion was that antenatal screening is the most cost-effective, feasible, and practical solution to the ongoing problem of congenital syphilis. It has the significant potential of reducing the prevalence of the disease and the adverse pregnancy outcomes associated with syphilis. The advent of newer rapid tests, including ICS, dual-POC, and the decentralized syphilis control programs incorporated into the universal screening program, has the potential to eliminate congenital syphilis. The summary of the conclusion with some of the recommendations in this regard is as follows. a) In Zambia and other resource-limited settings, a same-day test and treatment with penicillin should be prioritized to achieve the goal of eliminating congenital syphilis; b) universal screening of pregnant women in clinics can reduce the annual number of stillbirths, neonatal deaths, and incidence of congenital syphilis and can avert up to 2.6 million DALYs at an estimated annual direct medical cost of US$ 20.8 million; c) eliminating the MTCT of syphilis through screening and treatment in ANCs is highly cost-effective in a wide range of settings, especially in countries with a high prevalence; d) decentralization of antenatal screening with on-site confirmation, rapid point-of-care Treponema tests that do not require electricity, other laboratory equipment, or refrigeration, and can give results in 15 minutes can be valuable tools for preventing or eliminating congenital syphilis; e) following CDC recommendations, all pregnant women must be screened for syphilis at their first prenatal visit; and f) future analyses can be tailored to countries using local epidemiologic and programmatic data.
